# The impact of direct-to-consumer personal genomic testing on perceived risk of breast, prostate, colorectal, and lung cancer: findings from the PGen study

**DOI:** 10.1186/s12920-015-0140-y

**Published:** 2015-10-15

**Authors:** Deanna Alexis Carere, Tyler VanderWeele, Tanya A. Moreno, Joanna L. Mountain, J. Scott Roberts, Peter Kraft, Robert C. Green, for the PGen Study Group

**Affiliations:** Department of Epidemiology, Harvard School of Public Health, Boston, MA USA; Division of Genetics, Department of Medicine, Brigham and Women’s Hospital, EC Alumnae Building, Suite 301, 41 Avenue Louis Pasteur, Boston, MA 02115 USA; Pathway Genomics, San Diego, CA USA; 23andMe Inc., Mountain View, CA USA; Department of Health Behavior and Health Education, University of Michigan School of Public Health, Ann Arbor, MI USA; Harvard Medical School, Boston, MA USA; Partners Personalized Medicine, Boston, MA USA

## Abstract

**Background:**

Direct access to genomic information has the potential to transform cancer risk counseling. We measured the impact of direct-to-consumer genomic risk information on changes to perceived risk (ΔPR) of breast, prostate, colorectal and lung cancer among personal genomic testing (PGT) customers. We hypothesized that ΔPR would reflect directionality of risk estimates, attenuate with time, and be modified by participant characteristics.

**Methods:**

Pathway Genomics and 23andMe customers were surveyed prior to receiving PGT results, and 2 weeks and 6 months post-results. For each cancer, PR was measured on a 5-point ordinal scale from “much lower than average” to “much higher than average.” PGT results, based on genotyping of common genetic variants, were dichotomized as *elevated* or *average risk*. The relationship between risk estimate and ΔPR was evaluated with linear regression; generalized estimating equations modeled this relationship over time.

**Results:**

With the exception of lung cancer (for which ΔPR was positive regardless of result)*, elevated risk* results were significantly associated with positive ΔPR, and *average risk* results with negative ΔPR (e.g., prostate cancer, 2 weeks: least squares-adjusted ΔPR = 0.77 for *elevated risk* versus −0.21 for *average risk*; *p*-value_difference_ < 0.0001) among 1154 participants. Large changes were rare: for each cancer, <4 % of participants overall reported a ΔPR of ±3 or more units. Effect modification by age, cancer family history, and baseline interest was observed for breast, colorectal, and lung cancer, respectively. A pattern of decreasing impact on ΔPR over time was consistently observed, but this trend was significant only in the case of colorectal cancer.

**Conclusions:**

We have quantified the effect on consumer risk perception of returning genetic-based cancer risk information directly to consumers without clinician mediation. Provided via PGT, this information has a measurable but modest effect on perceived cancer risk, and one that is in some cases modified by consumers’ non-genetic risk context. Our observations of modest marginal effect sizes, infrequent extreme changes in perceived risk, and a pattern of diminishing impact with time, suggest that the ability of PGT to effect changes to cancer screening and prevention behaviors may be limited by relatively small changes to perceived risk.

**Electronic supplementary material:**

The online version of this article (doi:10.1186/s12920-015-0140-y) contains supplementary material, which is available to authorized users.

## Background

Direct-to-consumer personal genomic testing (PGT) is distinct from clinical genetic testing in both its process and goals: whereas clinical testing is typically ordered as part of a medical evaluation to identify highly penetrant mutations associated with rare phenotypes (e.g., Lynch syndrome), PGT provides generally healthy consumers with information about common single nucleotide polymorphisms (SNPs) that have been associated with multifactorial diseases (e.g., colorectal cancer). PGT is not diagnostic, but provides risk information to a consumer that he or she may consider in making health-related decisions, such as when to begin cancer screening (e.g., colonoscopy).

The possibility that PGT could impact health services has prompted professional organizations [1, 2], including the American Society of Clinical Oncology [3], to call for research in this area, and a considerable body of research on the consumer and physician experience of PGT now exists [4]. After nearly a decade of such research, however, PGT continues to be controversial [5–8], and in November 2013, the United States Food and Drug Administration (FDA) ordered 23andMe, Inc. [9] (23andMe)—a leading PGT provider—to cease marketing of their health-related PGT services. Since then, 23andMe’s health-related services have not been reinstated in the United States, but in 2014 the company launched similar services in both Canada [10] and the United Kingdom [11].

Here, we have analyzed the impact of PGT-derived cancer risk information on consumers’ perceived risk of four cancers for which screening is possible (breast, prostate, colorectal, and lung) using data from the Impact of Personal Genomics (PGen) Study [12]. To date, there have been no empirical studies focused specifically on PGT for cancer risk, despite the fact that it has the potential to alter the landscape of cancer genetic counseling by improving the precision of individual risk estimates [13, 14], increasing demand for cancer screening [15], and shifting control of genetic screening from physicians to consumers [16].

Due to the importance of perceived disease risk in numerous prominent theories of health behavior [17], we chose as the primary outcome measure in our analyses change in perceived risk (ΔPR) of each cancer from baseline (pre-results) to 2 weeks and 6 months post-results. We hypothesized that: ΔPR for each cancer would be significantly associated with PGT risk estimate at both follow-up time points; directionality of ΔPR would reflect direction of PGT risk estimate relative to average population risk; magnitude of ΔPR would attenuate with time; and the effect of PGT risk estimate on ΔPR would be modified by baseline participant characteristics.

## Methods

### The impact of Personal Genomics (PGen) study

The PGen Study is a collaboration between academic researchers and two PGT companies, and the academic-industry partnership [18] and recruitment and data collection methods [12] have been described in detail elsewhere. Briefly, new customers of 23andMe and Pathway Genomics [19] (Pathway) were recruited online after placing an order for direct-to-consumer PGT between March and July 2012. Following an online consent process, participants were invited to three web-based surveys administered by Survey Sciences Group, LLC (Ann Arbor, Michigan): at baseline, after they had ordered testing but prior to receiving their results (BL); 2 weeks after viewing their results (2 W); and 6 months after viewing their results (6 M). Results were returned to customers according to the standard practice of each company and were linked to survey data at the end of survey administration. In total, 1464 participants completed the baseline survey and were eligible for follow-up; of these, 1046 (71.4 %) and 1042 (71.2 %) submitted the 2-week and 6-month surveys, respectively. Institutional approval was obtained from the Partners Human Research Committee and the University of Michigan School of Public Health Institutional Review Board.

### Exposure variables

Participants received a genetic risk estimate, based on genotyping of multiple SNPs (Additional file 1: Table S1), for each of breast (women only), prostate (men only), colorectal and lung cancer. 23andMe customers were provided with a report that included: (1) a baseline, 10-year age-adjusted risk of each cancer, assigned prior to genetic testing; (2) an age-adjusted relative risk for each cancer based on the customer’s genetic profile; and (3) a revised 10-year age-adjusted risk of each cancer computed by multiplying together values (1) and (2). Risk estimates were additionally adjusted for biological sex in the case of colorectal cancer (Additional file 1: Table S2). These results were presented in the form of two diagrams each with 100 human figures, the first with a proportion shaded in to represent the general population risk (quantity 1, above), and the second with a proportion shaded in to represent the genetics-adjusted risk (quantity 3, above).

Pathway customers generally received results on a 5-category scale corresponding to increasing RR of disease; however, in the case of the four cancers being studied, all results provided were in either the second lowest category (*Learn More*: “Your genetic profile gives you an average predisposition to these conditions, and most people fall in this category. You should focus on disease prevention, learn about your family history and how lifestyle choices influence disease onset”) or the middle category (*Be Proactive*: “Your genetic profile shows increased susceptibility for these health conditions. You should make an effort to learn the warning signs, contributing lifestyle factors, and your family history for these conditions. Speak with your doctor about developing a prevention plan.”) (Additional file 1: Table S3).

In order to harmonize genetic risk information across companies, a threshold RR level was selected to distinguish *elevated* from *average* genetic risk. This process was undertaken during the data cleaning stage of the PGen Study and prior to any analysis of study data, including but not limited to the analysis presented here. Based upon consideration of their results reporting standards, each company advised PGen Study researchers on determination of this threshold. A threshold of RR ≥ 1.2 to distinguish elevated genetic risk was ultimately chosen based on three considerations: 1) 23andMe representatives indicated that the company generally considers average risk as within 20 % of general population risk; 2) Pathway representatives agreed that this threshold would in most instances match well the cut-point between their risk categories of “Learn More” and “Be Proactive”; and 3) PGen Study researchers agreed that in the context of DTC-PGT and genetic testing of common, low-penetrance variants, a RR ≥ 1.2 was appropriately indicative of an elevated genetic risk. Results across both companies were therefore dichotomized into two categories: *average genetic risk* (RR < 1.2; “Learn More” category) and *elevated genetic risk* (RR ≥ 1.2; “Be Proactive” category). Due to the restricted distribution of Pathway results, we were unable to discriminate in our analyses between *average* and *reduced* genetic risk results.

### Outcome variables

Participants were asked on all surveys to rate their chances of developing each type of cancer “compared to the average [man or woman] of [the same] age.” Responses were recorded on a 5-point risk perception scale ranging from *much lower than average* (1) to *much higher than average* (5); [20, 21] alternatively, participants could select “I have been diagnosed with this condition.” Perceived risk (PR) was operationalized as a continuous variable, with each step on the 5-point scale corresponding to a 1 unit change.

### Other variables

Age, race/ethnicity [22], gender, annual household income, highest education level, interest in cancer-specific PGT results (‘very interested,’ ‘somewhat interested,’ ‘not at all interested,’ for each cancer type), smoking status (‘never,’ ‘past,’ or ‘current’), and cancer family history were measured at baseline. Participants were asked to report on each blood relative with cancer, including both maternal and paternal families, and to indicate the type of cancer diagnosed in that relative. A series of conditional survey branches were used to obtain this information: e.g., (1) “Which of your blood relatives have ever had [cancer]?” (choose all that apply from list of relation types); (2) if “brother or sister” is selected, participant would be prompted with “Please select the type(s) of [cancer] that [a brother or sister] has had” (choose all that apply from a list of cancer types). Additional conditional branches were generated for each relative reported to have a history of cancer, but we did not collect information on age at diagnosis or family size. Using this data, a site-specific, 3-level ordinal family history variable was created for each of the four cancers, with levels corresponding to “no family history,” “family history in 2nd degree relative(s) only,” and “family history in 1st degree relative(s).” A general cancer family history variable, inclusive of all cancer types, was created in the same way. Use of cancer screening services since undergoing PGT was queried at 6 months with questions from the 2011 Behavioral Risk Factor Surveillance System Questionnaire, modified to reflect a 6-month window of interest [23].

### Statistical analyses

Data for this analysis were obtained from 1155 participants who completed the BL survey plus at least one follow-up survey. Primary analysis samples were restricted to participants with an available genetic risk estimate for the cancer being studied; no missing data for BL-, 2 W-, or 6 M-PR (as necessary) for that cancer; and no reported diagnosis of the cancer being studied at any time during the data collection period.

We first performed linear regressions of change in PR from BL to 2 W (ΔPR_2W_) and from BL to 6 M (ΔPR_6M_) for each cancer. Analyses were adjusted for BL-PR, age, gender, race (White vs. non-White), Hispanic/Latino ethnicity, education (4 categories), smoking status (lung cancer only), and testing company; cancer family history and interest in cancer-specific results were evaluated as possible confounders. From the resulting linear regression models, least squares-adjusted mean ΔPRs were computed, stratified by genetic risk estimate *(elevated risk* versus *average risk*).

We used generalized estimation equations (GEEs) to account for the expected correlation between ΔPR_2W_ and ΔPR_6M_ and to evaluate the hypothesis that the effect of genetic risk estimate on ΔPR varied by follow-up time. In each model we included an interaction term between follow-up survey time and genetic risk estimate, and used a Wald test of significance to evaluate our hypothesis.

We next investigated effect modification by baseline participant characteristics. In order to maximize power, we conducted interaction analyses in the relevant 2 W follow-up samples, which had the smallest amount of missing data, and used the corresponding linear regression model (described above). Wald tests of significance were used to evaluate, in turn, interaction terms between genetic risk estimate and each of the following: baseline interest, age, gender (for colorectal and lung cancers), cancer family history, and smoking status (lung cancer only). Significant interaction terms were retained, and a final regression model was obtained for each cancer. From these, least squares-adjusted mean ΔPRs, stratified by genetic risk estimate and significant effect modifiers, were computed.

### Sensitivity analyses

To assess the impact on our results of the decision to use linear regression modeling of ΔPR, which assumes a constant effect of genetic risk information on any 1-unit change in PR, we alternately performed each of the 2 W regression analyses using generalized logistic regression with ΔPR = 0 as the reference category for the outcome. To evaluate our selection of relative risk rather than absolute risk as the main predictor of ΔPR, we evaluated the correlation between RR and absolute risk results for each cancer. We also transformed RR into a four-category variable corresponding to quartiles (RRq), performed a linear trend test of RRq in the ΔPR_6M_ linear regression models for each cancer, and then evaluated RRq as a categorical variable to observe the pattern of effect across quartiles. Because absolute risk and RR values were not available for Pathway Genomics participants, they were excluded from these analyses.

To evaluate the possibility that ΔPR was affected by cancer screening undertaken as a result of PGT, we repeated the ΔPR_6M_ linear regressions, excluding anyone who had received screening for the relevant cancer since receiving their results. To evaluate the impact of informative censoring due to missing PR data at follow-up, we repeated the ΔPR_6M_ linear regressions on a pseudo-population created using inverse probability-weighting (IPW) for missing data [24].

All analyses were conducted using SAS software (version 9.3; SAS Institute, Cary, NC), and models were fitted using PROC GLM (linear regressions) and PROC GENMOD (longitudinal analyses and IPW). Statistical significance for all analyses was set at *p* < 0.05.

## Results

### Participants

One participant was excluded from all analyses due to missing gender data. For each cancer, between 6.2 % and 14.5 % of participants were excluded from analysis due to missing baseline PR, missing genetic risk data, or a cancer diagnosis prior to or during the data collection period. Sample sizes for each cancer-specific analysis are presented in Table 1.

**Table 1:** Sample sizes, stratified by analysis and cancer type

Sample definition	Breast (Women only)	Prostate (Men only)	Colorectal	Lung
Available survey responses	700	454	1154	1154
Eligible survey responses^a^	649	388	1082	1080
2 W linear regression^b^	576	354	969	966
6 M linear regression^c^	565	343	947	945
Longitudinal analysis^d^	500	314	847	844

*2 W* 2-week follow-up, *6 M* 6-month follow-up

^a^No missing data for cancer-specific genetic risk result or baseline perceived risk; no reported cancer-specific diagnosis during data collection period

^b^Eligible survey responses, with available data for 2 W perceived risk

^c^Eligible survey responses, with available data for 6 M perceived risk

^d^Eligible survey responses, with available data for both 2 W and 6 M perceived risk

Subjects reporting cancer-specific PRs were similar in most cases with respect to age, gender, education, income, race, smoking status, and general cancer family history (Table 2), although female participants reporting breast cancer PR reported significantly lower levels of education (X_3_^2^ = 8.2, *p* = 0.040) and income (X_4_^2^ = 20.7, *p* = 0.0004) and greater frequency of any cancer family history (X_3_^2^ = 8.2, *p* = 0.016) than male participants reporting prostate cancer PR. Baseline PR, interest in cancer-specific PGT results, and reported cancer-specific family history showed considerable variation across samples: for example, only 40.9 % of participants reporting lung cancer PR were ‘very interested’ in learning their genetic risk of lung cancer, while 66.6 % of participants reporting breast cancer PR were ‘very interested’ in learning their genetic risk of breast cancer.

**Table 2:** Characteristics of PGen study participants included in at least one linear regression analysis for change in perceived risk of cancer

	Breast (*n* = 641)		Prostate (*n* = 383)		Colorectal (*n* = 1069)		Lung (*n* = 1067)	
	No.	%	No.	%	No.	%	No.	%
Female gender	641	100.0	0	0.0	646	60.4	647	60.6
Non-white race	90	14.0	43	11.2	145	13.6	144	13.5
Hispanic/latino ethnicity	23	3.6	20	5.2	39	3.7	39	3.7
Highest level of education								
No college degree	152	23.7	64	16.7	224	20.9	223	20.9
College degree only	194	30.3	121	31.6	327	30.6	326	30.6
Some graduate school	219	34.2	139	36.3	375	35.1	375	35.1
Doctoral-level degree	76	11.8	59	15.4	143	13.4	143	13.4
Annual household income								
< $40,000	118	18.4	55	14.4	177	16.5	176	16.5
$40,000–$69,999	128	20.0	55	14.4	192	17.9	192	18.0
$70,000–$99,999	135	21.1	75	19.6	223	20.9	223	20.9
$100,000–$199,999	193	30.1	126	32.9	329	30.9	331	31.0
≥ $200,000	58	9.0	66	17.2	133	12.4	130	12.2
Not reported	9	1.4	6	1.5	15	1.4	15	1.4
Any cancer family history								
Affected FDR(s)	296	46.2	167	43.6	489	45.7	488	45.8
Affected SDR(s) only	219	34.2	116	30.3	345	32.3	345	32.3
No affected FDR/SDR(s)	116	18.1	99	25.8	223	20.9	222	20.8
Not reported	10	1.5	1	0.3	12	1.1	12	1.1
Specific cancer family history^a^								
Affected FDR(s)	84	13.1	29	7.6	72	6.8	65	6.1
Affected SDR(s) only	137	21.4	21	5.5	138	12.9	139	13.0
No affected FDR/SDR(s)	410	64.0	332	86.7	847	79.2	851	79.8
Not reported	10	1.5	1	0.2	12	1.1	12	1.1
Interest in cancer-specific PGT								
Not at all interested	37	5.8	19	5.0	120	11.2	209	19.6
Somewhat interested	177	27.6	136	35.5	392	36.7	421	39.5
Very interested	427	66.6	228	59.5	557	52.1	437	40.9
Pathway customers	290	45.2	115	30.0	415	38.8	414	38.8
Age, years								
Mean ± standard deviation	46.8 ± 14.9		45.7 ± 16.2		47.1 ± 15.7		47.1 ± 15.7	
Range	19, 92		19, 91		19, 94		19, 94	
Baseline perceived risk^b^								
Mean ± standard deviation	2.8 ± 1.0		2.9 ± 0.9		2.7 ± 1.0		2.3 ± 1.0	
Range	1, 5		1, 5		1, 5		1, 5	

*FDR* first-degree relative, *SDR* second-degree relative, *PGT* personal genomic testing

^a^Only includes reported cases of the type of cancer being studied

^b^For the specific cancer being studied, rated from “much below average” (1) to “much higher than average” (5)

### Change in perceived risk of cancer

*Elevated risk* results were least frequent for breast cancer (10.8 % in 2 W follow-up samples; 9.6 % in 6 M follow-up samples) and most frequent for colorectal cancer (24.9 %; 24.3 %) (Table 3). Compared to participants who received *elevated risk* results, those who received *average risk* results more frequently indicated no change in perceived risk of a particular cancer. In all cases, an increase in perceived risk of *x* units among participants who received *elevated risk* results was more common than a decrease in perceived risk of *x* units among participants who received *average risk* results. Changes to PR, stratified by cancer and genetic risk result, are shown in Table 3. Overall, changes of > 2 units (PR categories) were infrequent: at 2 W follow-up, 8 (1.4 %), 12 (3.4 %), 16 (1.7 %), and 19 (2.0 %) participants reported changes of ±3 or ±4 units for breast, prostate, colorectal, and lung cancer, respectively. At 6 M follow-up, these same values were 9 (1.6 %), 8 (2.3 %), 8 (0.8 %), and 24 (2.5 %).

**Table 3:** Distribution of changes in perceived risk from baseline to follow-up, stratified by cancer, genetic result, and follow-up time

	Breast		Prostate		Colorectal		Lung	
Genetic risk result	Average	Elevated	Average	Elevated	Average	Elevated	Average	Elevated
2-week follow-up								
Frequency, *n* (%)	514 (89.2)	62 (10.8)	278 (78.5)	76 (21.5)	728 (75.1)	241 (24.9)	781 (80.8)	185 (19.2)
Unit change in perceived risk (%)								
−4	0.0	0.0	0.0	0.0	0.1	0.0	0.1	0.0
−3	0.6	0.0	1.4	0.0	0.7	0.4	0.5	0.0
−2	6.8	6.5	5.0	1.3	3.6	0.8	2.7	1.6
−1	23.7	6.5	26.6	14.5	22.7	11.6	19.1	11.4
0	51.6	37.1	51.1	25.0	50.0	38.2	47.0	37.3
+ 1	14.0	35.5	14.0	35.5	19.2	35.7	24.2	31.9
+ 2	3.3	14.5	1.4	14.5	3.7	9.5	5.9	12.4
+ 3	0.0	6.5	0.4	7.9	0.0	3.7	0.5	5.4
+ 4	0.0	0.0	0.0	1.3	0.0	0.0	0.0	0.0
6-month follow-up								
Frequency, *n* (%)	511 (90.4)	54 (9.6)	266 (77.6)	77 (22.4)	717 (75.7)	230 (24.3)	773 (81.8)	172 (18.2)
Unit change in perceived risk (%)								
−4	0.0	0.0	0.0	0.0	0.0	0.0	0.1	0.0
−3	0.6	1.9	0.8	0.0	0.1	0.0	0.7	0.0
−2	6.7	0.0	4.9	5.2	4.6	1.7	2.7	1.7
−1	23.1	18.5	26.3	10.4	21.5	15.7	18.4	12.8
0	50.9	33.3	51.5	32.5	52.4	40.9	48.0	41.3
+1	13.9	27.8	14.7	33.8	16.9	30.0	22.9	27.9
+2	4.5	13.0	1.9	10.4	4.2	9.6	6.3	10.5
+3	0.4	1.9	0.0	5.2	0.1	2.2	0.8	5.8
+4	0.0	3.7	0.0	2.6	0.1	0.0	0.1	0.0

### Change in perceived risk and genetic risk results

At both follow-up time points, and for all four cancers, multivariate linear regression revealed a significant effect of genetic risk estimate on ΔPR (Table 4). In general, mean ΔPR among those receiving an *elevated risk* result (range = 0.33–0.77 units) was greater in magnitude than mean ΔPR among those receiving an *average risk* result (range = 0.04–0.22 units). Mean ΔPR for lung cancer was positive, regardless of genetic risk estimate, at both time points. For all other cancers, mean ΔPR was positive among those receiving an *elevated risk* result and negative among those receiving an *average risk* result. Effect estimates did not change in models that included adjustment for general cancer family history, specific cancer family history, or baseline interest in PGT cancer risk results.

**Table 4:** Linear regression and generalized estimating equation models for effect of genetic risk estimate on change in perceived risk of cancer

	Breast	Prostate	Colorectal	Lung^c^
Change in perceived risk: baseline to 2W^a^				
Elevated risk result: LS mean (95 % CI)	0.61 (0.42, 0.79)	0.77 (0.58, 0.95)	0.50 (0.41, 0.60)	0.62 (0.49, 0.75)
Average risk result: LS mean (95 % CI)	−0.20 (−0.27, −0.14)	−0.21 (−0.31, −0.11)	−0.05 (−0.10, 0.01)	0.18 (0.10, 0.27)
LS mean difference (95 % CI)	0.81 (0.62, 1.00)	0.97 (0.76, 1.19)	0.55 (0.44, 0.66)	0.44 (0.31, 0.57)
*p*-value_Difference_	<0.0001	<0.0001	<0.0001	<0.0001
Change in perceived risk: baseline to 6M^a^				
Elevated risk result: LS mean (95 % CI)	0.53 (0.32, 0.74)	0.51 (0.32, 0.69)	0.33 (0.24, 0.43)	0.58 (0.44, 0.72)
Average risk result: LS mean (95 % CI)	−0.15 (−0.22, −0.07)	−0.14 (−0.24, −0.04)	−0.04 (−0.09, 0.02)	0.22 (0.13, 0.31)
LS mean difference (95 % CI)	0.68 (0.46, 0.90)	0.65 (0.43, 0.86)	0.37 (0.26, 0.48)	0.36 (0.22, 0.49)
*p*-value_Difference_	<0.0001	<0.0001	<0.0001	<0.0001
GEE Model: ΔPR^b^				
Elevated risk result: β ± SE (*p*-value)	0.68 ± 0.29 (0.02)	1.06 ± 0.20 (<0.0001)	0.79 ± 0.11 (<0.0001)	0.59 ± 0.13 (<0.0001)
Elevated risk result*survey: β ± SE (*p*-value)	0.06 ± 0.19 (0.76)	−0.15 ± 0.11 (0.17)	−0.20 ± 0.07 (0.0027)	−0.11 ± 0.08 (0.17)

*2 W* 2-week follow-up, *6 M* 6-month follow-up, *LS* least squares adjusted, *CI* confidence interval, *SE* standard error

^a^Adjusted for baseline perceived risk, age, gender, race/ethnicity, education, and company

^b^Adjusted for baseline perceived risk, age, gender, race/ethnicity, education, and company, with result*survey interaction term

^c^Multivariate lung cancer analyses additionally adjusted for smoking status

A trend of greater effect sizes at 2 W follow-up compared to 6 M follow-up was apparent for all cancers; however, GEE modeling of the survey by genetic risk estimate interaction found evidence for significant effect modification by follow-up time only in the case of colorectal cancer (Table 4).

### Effect modification

Increasing age was associated with a significantly attenuated effect of genetic risk estimate on ΔPR of breast cancer at 2-week follow-up (p_interaction_ = 0.0467). Among women in the lowest age group by decile, an *elevated risk* result was associated with a 1.12 unit greater ΔPR than an *average risk* result (95 % CI = 0.78, 1.46). For each decile increase in age category, this difference decreased by an average of 0.08 units (0.007, 0.14). A similar trend was observed for prostate cancer, but the interaction term between risk result and age was non-significant (*p* = 0.0591).

**Fig. 1:**
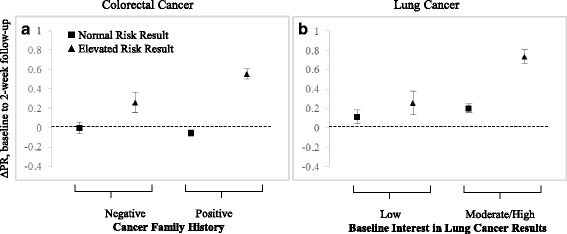
Modifiers of the effect of genetic risk estimate on change in perceived risk (ΔPR) of cancer. **a**. Mean ΔPR of colorectal cancer, stratified by cancer family history status. An *average risk* result was associated with a non-significant mean ΔPR of −0.002 (95 % CI = −0.12, 0.11) in participants reporting no family history of cancer, and a non-significant mean ΔPR of −0.05 (−0.12, 0.007) in those reporting a positive family history. An *elevated risk* result was associated with a mean ΔPR of 0.26 (0.06, 0.47) in the absence of family history, and a mean ΔPR of 0.56 (0.45, 0.66) in the presence of a family history (p_interaction_ = 0.0093). **b**. Mean ΔPR of lung cancer, stratified by baseline interest in lung cancer risk results. An *average risk* result was associated with a non-significant mean ΔPR of 0.11 (−0.03, 0.25) in participants who expressed low interest in lung cancer risk information at baseline, and a mean ΔPR of 0.20 (0.11, 0.29) in those who expressed a moderate or high interest in this information at baseline. An *elevated risk* result was associated with a mean ΔPR of 0.26 (0.01, 0.50) given low interest, and a mean ΔPR of 0.73 (0.59, 0.88) given moderate or high interest (p_interaction_ = 0.0434). Error bars represent standard error of the mean

Family history of cancer was a significant modifier of the effect of genetic risk estimate on ΔPR of colorectal cancer at 2-week follow-up (p_interaction_ = 0.0093). Among participants who reported no history of cancer in first- or second-degree relative, an *elevated risk* result was associated with a 0.26 unit greater ΔPR than an *average risk* result (95 % CI = 0.03, 0.50); however, among participants reporting a positive family history of cancer, an *elevated risk* result was associated with a 0.61 unit greater ΔPR than an *average risk* result (95 % CI = 0.49, 0.73). Adjusted mean ΔPR values, stratified by family history status and genetic risk result are shown in Fig. 1a.

Baseline interest in lung cancer genetic risk information was a significant modifier of the effect of genetic risk estimate on ΔPR of lung cancer (*p* = 0.0108). Among participants who indicated low interest in these results at baseline, there was no significant difference in the impact of an *elevated risk* result and an *average risk* result on ΔPR of lung cancer (mean difference = 0.14, 95 % CI = −0.12, 0.41). An *elevated risk* result was, however, associated with a significantly greater ΔPR than an *average risk* result among those participants who indicated a moderate or high interest in these results at baseline (mean difference = 0.53, 95 % CI = 0.39, 0.67). (“Moderate” and “High” interest categories were collapsed for presentation of the data because effect estimates and confidence intervals were identical in these two groups.) Adjusted mean ΔPR values, stratified by interest and genetic risk result are shown in Fig. 1b. There was no evidence of effect modification by smoking status; however, we noted a strong correlation between smoking status (never/former/current) and baseline interest in lung cancer genetic risk information (low/moderate/high), with 58 % of current smokers, 50 % of former smokers, and 34 % of never smokers indicating high interest in these results (X_2_^2^ = 28.9, *p* < 0.0001).

### Sensitivity analyses

The results of modeling ΔPR as a categorical variable are presented in Additional file 1: Table S4. The small number of people who reported large changes in PR made odds ratio estimates unstable for these categories (ΔPR > +2 or < −2) unstable; however, the results of using generalized logistic regression were qualitatively consistent with the results from our main linear regression models. A clear pattern of greater odds of an increase in PR—and lower odds of a decrease in PR—with an *elevated risk* result was observed, and these effects were significant across all cancers in the categories of ΔPR = +1 and ΔPR = +2, and across breast, colorectal, and lung cancer for the ΔPR = −1 category.

All 23andMe participants of the same sex were assigned the same cancer-specific population risk values; therefore, absolute risk and RR values were perfectly correlated after stratification by sex. Among 23andMe participants, the trend of effect across quartiles of RR suggested that the use of RR > 1.2 as the threshold for increased genetic risk was appropriate. For lung cancer, the effect of a *reduced risk* estimate was not significantly different from that of an *average risk* estimate; however, a significant difference in ΔPR between those who received a *reduced risk* estimate and those who received an *average risk* estimate was observed for breast, prostate, and colorectal cancer (Additional file 1: Table S5).

Among participants in the ΔPR_6M_ analyses, 311 (55.0 % of 565) reported some type of breast cancer screening (mammography, breast MRI, or clinical breast exam) since receiving their PGT results; 78 (22.7 % of 343) reported PSA testing; 87 (9.1 % of 947) reported a colonoscopy; and 14 (1.5 % of 945) reported lung cancer screening. Excluding screened participants from the linear regressions presented in Table IV made no substantial difference to the effect estimates or mean ΔPRs (Additional file 1: Table S6).

Missing data for PR-6 M resulted in censoring of 11.6–12.9 % of participants from each ΔPR_6M_ linear regression sample. IP weighting to adjust for this missing data revealed no significant differences in effect estimates (Additional file 1: Table S6).

## Discussion

We have shown that commercial PGT risk estimates based on common, low-penetrance SNPs have a modest but significant effect on perceived risk of breast, prostate, colorectal, and lung cancers. Directionality of effect was typically as expected, with *elevated risk* results corresponding to an increase in perceived cancer risk, and *average risk* results corresponding to unchanged or slightly lower perceived risk. Lung cancer proved exceptional in this respect: regardless of PGT result, we observed a mean increase in perceived risk of lung cancer following PGT. One explanation for this finding is that consumers may not have known, prior to undergoing PGT, that lung cancer risk includes not only an environmental component, but also a genetic component. Receiving a genetic-based risk prediction for lung cancer, then, may have suggested to consumers that they were at risk for lung cancer even if they did not have typical risk factors, such as smoking history and occupational exposures.

On the other hand, stratification by baseline interest revealed that the effect of genetic risk information for lung cancer was significantly greater among consumers who expressed (prior to receiving their results) an interest in obtaining this particular information about themselves than among consumers who were “not at all interested” in learning their genetic risk of lung cancer. Although there was no statistical evidence of effect modification by smoking status, we did observe a strong positive correlation between baseline interest in lung cancer genetic risk information and smoking history. Thus, one interpretation of the observed effect modification by interest is that smoking (or other carcinogenic exposure) history drives baseline interest in lung cancer genetic risk information, which in turn modifies consumers’ responses to receiving this information.

These findings highlight a complex relationship between genetic information and risk perception, and suggest that consumers incorporate both genetic and non-genetic risk factors into their personal estimation of risk. This interpretation is further supported by the results of effect modification analyses for breast and colorectal cancer: Older women, in whom the impact of genetic risk information was attenuated compared to younger women, have more information on which to base their perceived risk of breast cancer (e.g., results of prior breast screening), and may be aware that non-genetic factors (e.g., age) are more important components of their risk than the information provided by PGT. Conversely, in the case of colorectal cancer, for which the impact of genetic risk information was greater among those consumers with a positive family history of cancer than those without, the presence of a family history of cancer may lead consumers to put greater stock in their PGT results, since they already have independent evidence of “genetic risk” of cancer.

The relationship between genetic information and risk perception is likely further complicated by the method in which this information is communicated to consumers. Here, each company presented risk information in a different way: for example, 23andMe relied on a visual representation of risk as a proportion of persons, while Pathway presented consumers with a color-coded hierarchy of risk categories. These differences in risk presentation are not expected to lead to confounding after adjustment for PGT company; however, it is possible that the effect of a genetic risk estimate on perceived risk could be attenuated—or magnified—by the way in which the risk is described to the consumer. Similarly, the choice of perceived risk scale may impact the magnitude of effect observed. If, for example, participants were asked to report their perceived risk as a percentage from 0 to 100, an analysis of change in perceived risk might have greater precision compared to our analysis, where perceived risk was measured on a 5-point scale.

Elucidating the relationship between genetic risk information and perceived risk may represent one step towards understanding the link between genetic risk information and health behavior changes, and commercial PGT presents an opportunity to study this relationship before SNP-based risk prediction moves into the clinic. PGT for cancer is particularly well-suited to such study because of broad public awareness of cancer risk factors [25] and prevention strategies [26], and the potential for PGT to impact demand for cancer screening services.

Perceived risk has been studied extensively in the context of clinical genetic testing for high-penetrance, Mendelian cancer syndromes [27, 28]. In this setting, findings include: higher perceived risk and increased colorectal cancer screening rates following a positive genetic test for Lynch syndrome; [29] lower perceived risk in non-carriers compared to carriers after testing for Lynch syndrome and Hereditary Breast and Ovarian Cancer syndrome; [30, 31] and attenuation of effect on perceived risk at long-term follow-up [27]. Our work, however, marks the first comprehensive study of perceived risk of cancer in the context of PGT, which differs from clinical genetic testing with respect to the genetic data on which results are based, how results are provided (online vs. discussion with a clinician) and to whom results are given (the general population vs. high-risk clinic patients). Of note, in our study of a non-clinical population, cancer family history and baseline interest in cancer risk results (each of which are likely to be stronger in high-risk clinic patients) were not significant predictors of change in perceived risk, and adjustment for these factors did not impact estimates of the effect of genetic risk information on perceived risk.

The question of how consumers *ought* to respond to their PGT results remains unresolved: [32] critics of PGT note that although the SNPs evaluated by PGT are validated with respect to disease association at the population level, their clinical utility is unknown [33], and computed risk estimates are calculated in different ways by different PGT companies [34]. If PGT-derived risk estimates inappropriately stimulate surveillance, then PGT could lead to unnecessary use of health services and iatrogenic complications [15]. Of note here is that large changes to perceived risk (±3 or more units) were infrequent in our study, suggesting that PGT may have limited potential to prompt meaningful health behavior changes (either positive or negative). Moreover, we observed a pattern of attenuated effect over time, a finding consistent with previous studies of perceived risk in the clinical genetics context (see previous paragraph and Senay and Kaphingst’s review of “anchoring-and-adjustment bias” [35]). This attenuation of effect—significant in the case of colorectal cancer, and non-significant but particularly suggestive in the smaller breast and prostate cancer samples—leaves open the possibility that perceived cancer risk among PGT consumers may ultimately revert to pre-PGT levels. If PGT is to prompt substantial changes to use of cancer screening services, our findings suggest that these changes may be most likely to occur shortly following PGT, and will become increasingly less likely with time since testing; analyses of PGen Study data are currently underway to investigate the impact of PGT on cancer screening.

Strengths of our study include its large sample size, consistency of results across four cancers, and robustness of results in sensitivity analyses. Limitations include those inherent to voluntary survey data, including the potential for selection bias. Analyses of effect modification were limited in power due to sample stratification; thus, response to PGT could be further influenced by baseline participant characteristics in ways that are not evident here. We were also limited in our ability to investigate the impact of receiving a genetic risk estimate indicating lower than average cancer risk, although analysis of the 23andMe data alone suggests that such risk information may be associated with a decrease in perceived cancer risk. Finally, our findings are generalizable to consumers obtaining PGT through a direct-to-consumer model, but not to other forms of genetic testing. PGen Study participants tended to be well-educated, high-earning, and White; interpretation of genetic risk information, and its impact on risk perception, may differ in groups without these qualities.

## Conclusions

SNP-based genetic risk information for four common types of cancer, when provided directly to consumers via commercial PGT, has a measurable effect on consumers’ perceived risk of these cancers. However, while perceived risk is affected in predictable ways at a population level, effect modification analyses suggest a complex interplay between genetic and non-genetic risk information in consumers’ estimation of personal risk. Our findings further suggest that the effect of PGT on use of cancer screening services and modification of risk behaviors may be mitigated by the small magnitude of its effect on risk perception.

## Additional file

Additional file 1: Table S1. SNPs included in commercial personal genomic testing panels for cancer risk assessment in July 2012. **Table S2.** Distribution of genetic risk estimates returned to 23andMe customers in the PGen Study. **Table S3.** Distribution of genetic risk estimates returned to Pathway Genomics customers in the PGen Study. **Table S4.** Sensitivity analyses: characterization of the outcome. **Table S5.** Sensitivity analyses: characterization of genetic risk results. **Table S6.** Sensitivity analyses: screening behaviors and missing data. (PDF 461 kb)

## Abbreviations

BL: Baseline
DTC: Direct-to-consumer
GEE: Generalized estimating equation
IP: Inverse probability
PGT: Personal genomic testing
PR: Perceived risk
RR: Relative risk
SNP: Single nucleotide polymorphism
2 W: 2-week
6 M: 6-month
