# Supplementary material for: The impact of direct-to-consumer personal genomic testing on perceived risk of breast, prostate, colorectal, and lung cancer: findings from the PGen study
Source: BMC Med Genomics. 2015 Oct 15;8:63. doi: 10.1186/s12920-015-0140-y (PMC4606558; doi:10.1186/s12920-015-0140-y)
Supplement: Additional file 1: Table S1. — SNPs included in commercial personal genomic testing panels for cancer risk assessment in July 2012. Table S2. Distribution of genetic risk estimates returned to 23andMe customers in the PGen Study. Table S3. Distribution of genetic risk estimates returned to Pathway Genomics customers in the PGen Study. Table S4. Sensitivity analyses: characterization of the outcome. Table S5. Sensitivity analyses: characterization of genetic risk results. Table S6. Sensitivity analyses: screening behaviors and missing data. (PDF 461 kb) [file 12920_2015_140_MOESM1_ESM.pdf]

The impact of personal genomic testing on perceived risk of breast, prostate, colorectal, and lung cancer: Findings from the PGen Study

Carere et al.

Supplemental Materials

Table S1. SNPs included in commercial personal genomic testing panels for cancer risk assessment in July 2012

Table S2. Distribution of genetic risk estimates returned to 23andMe customers in the PGen Study

Table S3. Distribution of genetic risk estimates returned to Pathway Genomics customers in the PGen Study

Table S4. Sensitivity analyses: characterization of the outcome

Table S5. Sensitivity analyses: characterization of genetic risk results

Table S6. Sensitivity analyses: screening behaviors and missing data

Table S1. SNPs included in commercial personal genomic testing panels for cancer risk assessment in July 2012

Cancer Site	Reference SNP Cluster ID	Chromosomal Locus	Mapped Gene[a]	MAF[b]	Odds Ratio[c]
**Breast**					
23andMe Only					
	rs4973768	3p24.1	*SLC4A7*	0.442	1.16
	rs9485370	6q25.1	NA	0.201	NA
	rs7222197	17q22	NA	0.319	NA
Pathway Genomics Only					
	rs10941679	5p12	*FGF10-AS1 – MRPS30*	0.238	1.13
	rs889312	5q11.2	*RPL26P19 – MAP3K1*	0.300	1.13
	rs2046210	6q25.1	*CCDC170 – ESR1*	0.288	1.29
	rs6964587	7q21.2	NA	0.388	NA
	rs13281615	8q24.21	*LOC101930033*	0.454	1.08
	rs2981578	10q26.13	*FGFR2*	0.485	1.23
	rs12184413	11q22.2	NA	0.102	NA
	rs3817198	11p15.5	*LSP1; LOC102724536*	0.327	1.07
	*1592delT**	16p12.2	*PALB2*	-	-
Both Companies					
	rs13387042	2q35	*TNP1 – DIRC3*	0.438	1.2
	rs1045485	2q33.1	NA	0.123	NA
	rs1219648	10q26.13	*FGFR2*	0.465	1.2
	rs3803662	16q12.1	*CASC16*	0.248	1.28
	1100delC[d]	22q12.1	*CHEK2*	-	-
**Prostate**					
23andMe Only					
	rs12621278	2q31.1	*ITGA6*	0.040	1.33
	rs17021918	4q22.3	*PDLIM5*	0.354	1.11
	rs1512268	8p21.2	*FAM60DP – NKX3-1*	0.420	1.18
	rs10505483	8q24.21	*SRRM1P1 – CCAT1*	0.031	1.73
	rs7127900	11p15.5	*MIR4686 – ASCL2*	0.235	1.22
	rs4430796	17q12	*HNF1B*	0.491	1.22
	rs8102476	19q13.2	*DPF1 – PPP1R14A*	0.496	1.12
Pathway Genomics Only					
	rs5945572	Xp11.22	*CXorf67 – NUDT11*	0.426	1.23
	rs721048	2p15	*EHBP1*	0.137	1.15
	rs2660753	3p12.1	*PPATP1 – LINC00506*	0.102	1.18
	rs401681	5p15.22	*CLPT1ML*	0.434	NA
	rs9364554	6q25.3	*SLC22A3*	0.274	1.17
	rs6465657	7q21.3	*LMTK2*	0.491	1.12
	rs10090154	8q24.21	*CASC8 – CASC11*	0.055	1.68
	rs6983561	8q24.21	*SRRM1P1 – CCAT1*	0.023	1.87
	rs1571801	9q33.3	NA	0.290	NA
	rs4962416	10q26.13	*CTBP2*	0.252	1.17
	rs7931342	11q13.3	*MIR3164 – MYEOV*	0.469	1.19
	rs7501939	17q12	*HNF1B*	0.434	1.41
	rs9623117	22q13.1	*TNRC6B*	0.221	1.18
Both Companies					
	rs10486567	7p15.2	*JAZF1*	0.248	1.12

	rs1447295	8q24.21	*CASC8*	0.071	1.43
	rs6983267	8q24.21	*CCAT2; CASC8*	0.487	1.26
	rs10993994	10q11.23	*MSMB*	0.341	1.16
	rs1859962	17q24.3	*CASC17*	0.473	1.2
**Colorectum**					
Pathway Genomics Only					
	rs16892766	8q23.3	*LINC00536 – EIF3H*	0.106	1.27
	rs10795668	10p14	*RNA5SP299 – LINC00709*	0.326	1.12
	rs4444235	14q22.2	*BMP4*	0.442	1.11
	rs9929218	16q22.1	*CDH1*	0.295	1.1
	rs10411210	19q13.11	*RHPN2*	0.084	1.15
	rs961253	20p12.3	*FGFR3P3 – CASC20*	0.403	1.12
Both Companies					
	rs6983267	8q24.21	*CCAT2; CASC8*	0.487	1.27
	rs3802842	11q23.1	*COLCA2; COLCA1*	0.235	1.11
	rs4779584	15q13.3	*SCG5 – GREM1*	0.168	1.23
	rs4939827	18q21.1	*SMAD7*	0.473	1.16
**Lung**					
23andMe Only					
	rs8034191	15q25.1	*HYKK*	0.418	1.3
Pathway Genomics Only					
	rs2736100	5p15.33	*TERT*	0.473	1.12
	rs3117582	6p21.33	*BAG6; APOM*	0.080	1.24
	rs1051730	15q25.1	*CHRNA3*	0.385	1.35

Abbreviations: GWAS, genome-wide association study; MAF, minor allele frequency; NA, not available; rsID, reference SNP cluster ID; SNP, single nucleotide polymorphism

[a] Genes mapped to the strongest SNP. If the SNP is located within a gene, that gene is listed. If the SNP is intergenic, the upstream and downstream genes are listed, separated by a hyphen. Source: National Human Genome Research Institute Catalog of Published GWAS (http://www.genome.gov/26525384)

[b] Minor allele frequency reported in Utah residents from Northern and Western European ancestry from the CEPH collection. Source: International HapMap Project, Genome Browser release #28 (http://hapmap.ncbi.nlm.nih.gov/)

[c] From the 1st published GWAS on the association between the SNP and the relevant cancer listed in the NHGRI Catalog of Published GWAS.

[d] Rare variants (MAF < 0.02), for which only the mutation, location, and gene are provided.

Table S2. Distribution of genetic risk estimates returned to 23andMe customers in the PGen Study

	Breast n = 351	Prostate n = 268	Colorectal n = 654	Lung n = 653
Baseline 10-year Absolute Risks[a]				
Estimate (n)	0.09 (2)<br>0.14 (349)	0.11 (2)<br>0.18 (266)	0.03 (2)[b]<br>0.04 (362)[b]<br>0.05 (2)[c]<br>0.06 (288)[c]	0.06 (365)<br>0.08 (288)
Genetics-adjusted Relative Risk[e]				
Mean ± SD	0.98 ± 0.24	1.08 ± 0.42	1.05 ± 0.23	0.95 ± 0.17
Range	0.52 – 1.80	0.19 – 2.34	0.62 – 1.92	0.81 – 1.38
$10^{th}/90^{th}$ Percentile	0.70 / 1.33	0.60 / 1.69	0.75 / 1.37	0.81 / 1.37
Genetics-adjusted 10-year Absolute Risk[d]				
Mean ± SD	0.13 ± 0.03	0.19 ± 0.08	0.05 ± 0.01	0.07 ± 0.02
Range	0.07 – 0.24	0.03 – 0.42	0.02 – 0.10	0.05 – 0.12
$10^{th}/90^{th}$ Percentile	0.09 / 0.18	0.11 / 0.30	0.03 / 0.07	0.05 / 0.09

[a] Baseline 10-year risks were assigned to each consumer by 23andMe prior to undergoing genetic testing and are age-adjusted but not adjusted for competing risks.
[b] Female participants (estimates conditioned on biological sex)
[c] Male participants (estimates conditioned on biological sex)
[d] Genetics-adjusted risks are also age-adjusted (and conditioned on biological sex in the case of colorectal cancer) but are not adjusted for competing risks.

Table S3. Distribution of cancer genetic risk estimates returned to Pathway Genomics customers in the PGen Study

	Breast<br>n = 290	Prostate<br>n = 115	Colorectal<br>n = 415	Lung<br>n = 414
Learn More / Average Risk	280 (96.6%)	114 (99.1%)	319 (76.9%)	293 (70.8%)
Take Action / Increased Susceptibility	10 (3.5%)	1 (0.87%)	96 (23.1%)	121 (29.2%)

Table S4. Sensitivity analyses: characterization of the outcome

	Breast (n = 576)		Prostate (n = 354)		Colorectal (n = 969)		Lung[a] (n = 966)	
ΔPR-2W	Odds Ratio[a] (95% CI)	p-value	Odds Ratio[a] (95% CI)	p-value	Odds Ratio[a] (95% CI)	p-value	Odds Ratio	p-value
-4	n/a[c]	--	n/a	--	1.70 (<0.001, >999.99)	0.99	2.10 (<0.001, >999.99)	0.95
-3	<0.001 (<0.001, >999.99)	0.89	0.006 (<0.001, >999.99)	0.91	0.35 (0.02, 3.64)	0.38	<0.001 (<0.001, >999.99)	0.94
-2	0.41 (0.10, 1.58)	0.19	0.19 (0.02, 1.71)	0.14	0.09 (0.02, 0.46)	0.0037	0.45 (0.12, 1.69)	0.24
-1	0.18 (0.05, 0.58)	0.0041	0.69 (0.28, 1.7)	0.42	0.47 (0.28, 0.78)	0.0034	0.55 (0.31, 0.97)	0.0391
0	Ref.	--	Ref.	--	Ref.	--	Ref.	--
+1	14.3 (5.8, 35.2)	< 0.0001	9.67 (3.97, 23.5)	<.0001	3.78 (2.51, 5.70)	<.0001	1.99 (1.30, 3.06)	0.0017
+2	24.3 (4.43, 133.42)	0.0002	83.7 (14.2, 494.2)	<.0001	9.81 (4.49, 21.4)	<.0001	4.22 (2.15, 8.27)	<.0001
+3	>999.99 (29.2, >999.99)	0.0014	>999.99 (2.89, >999.99)	0.0342	>999.99 (<0.001, >999.99)	0.55	31.37 (6.79, 144.82)	<.0001
+4	n/a	--	>999.99 (<0.001, >999.99)	0.67	n/a	--	n/a	--

Abbreviations: CI, confidence interval; n/a, not applicable; Ref., reference category; ΔPR-2W, change in perceived risk from baseline to 2-week follow-up

[a] Results from multivariate generalized logistic regression for change in perceived risk from baseline to 2-week follow-up, adjusted for baseline perceived risk, age, gender, race/ethnicity, education, and company

[b] Multivariate lung cancer analyses additionally adjusted for smoking status.

[c] Results marked n/a indicate that no participants reported a change in perceived risk of this magnitude

Table S5. Sensitivity analyses: characterization of genetic risk results

	Breast (n = 310)		Prostate (n = 243)		Colorectal (n = 581)		Lung (n = 579)	
	β±SE[a] (p-value)	Mean RR±SD	β±SE[a] (p-value)	Mean RR±SD	β±SE[a] (p-value)	Mean RR±SD	β±SE[a,b] (p-value)	Mean RR±SD
**Dichotomous**								
RR≤1.2	---	0.90±0.16	---	0.85±0.23	---	0.95±0.15	---	0.90±0.08
RR>1.2	0.76±0.11 (<.0001)	1.37±0.13	0.98±0.12 (<.0001)	1.57±0.31	0.52±0.07 (<.0001)	1.36±0.16	0.47±0.10 (<.0001)	1.38±0.005
**4-Category (Quartiles)**								
Quartile 1	-0.23±0.12 (0.06)	0.71±0.07	-0.62±0.14 (<.0001)	0.60±0.24	-0.23±0.08 (0.0059)	0.78±0.08	-0.03±0.07 (0.68)	0.81±0.002
Quartile 2	---	0.88±0.04	---	0.89±0.06	---	1.00±0.06	---	0.97±0.001
Quartile 3	-0.01±0.13 (0.92)	1.03±0.06	0.18±0.14 (0.20)	1.13±0.08	0.004±0.09 (0.96)	1.12±0.06	0.45±0.10 (<.0001)	1.38±0.005
Quartile 4	0.45±0.12 (0.0003)	1.30±0.14	0.87±0.15 (<.0001)	1.65±0.29	0.45±0.08 (<.0001)	1.36±0.16	n/a[c]	n/a[c]
Trend Test	0.21±0.04 (<.0001)	0.98±0.24	0.46±0.05 (<.0001)	1.07±0.42	0.21±0.03 (<.0001)	1.06±0.24	0.18±0.05 (0.0003)	0.96±0.17

Abbreviations: RR, relative risk; SD, standard deviation; ΔPR_{2W}, change in perceived risk from baseline to two weeks post-results

[a] Results from multivariate linear regression for change in perceived risk from baseline to 2-week follow-up, adjusted for baseline perceived risk, age, gender, race/ethnicity, education, and company

[b] Multivariate lung cancer analyses additionally adjusted for smoking status.

[c] Due to the structure of the data, lung cancer genetic risk estimates could not be divided into quartiles, and were instead analyzed as tertiles.

Table S6. Sensitivity analyses: screening behaviors and missing data

	Breast	Prostate	Colorectal	Lung[d]
Multivariate Linear Regression[a], No Screeners[b]: Change in Perceived Risk: Baseline to 6M	n = 254	n = 265	n = 860	n = 931
Elevated Risk Result: LS Mean (95% CI)	0.62 (0.30, 0.93)	0.62 (0.40, 0.84)	0.32 (0.22, 0.42)	0.58 (0.44, 0.72)
Average Risk Result: LS Mean (95% CI)	-0.20 (-0.08, -0.32)	-0.11 (-0.23, -0.002)	-0.03 (-0.08, 0.03)	0.22 (0.14, 0.31)
LS Mean Difference (95% CI)	0.82 (0.49, 1.14)	0.74 (0.49, 0.98)	0.35 (0.23, 0.46)	0.36 (0.23, 0.50)
p-value$_{Difference}$	<.0001	<.0001	<.0001	<.0001
Multivariate Linear Regression[a], IPW for Censoring[c] Change in Perceived Risk: Baseline to 6M				
Censored Participants, n (%)	84 (12.9)	45 (11.6)	135 (12.5)	135 (12.5)
Non-censored Participants, n (%)	565 (87.1)	343 (88.4)	947 (87.5)	945 (87.5)
Elevated Risk Result: LS Mean (95% CI)	0.53 (0.24, 0.82)	0.51 (0.27, 0.74)	0.33 (0.23, 0.44)	0.57 (0.42, 0.73)
Average Risk Result: LS Mean (95% CI)	-0.15 (-0.08, -0.21)	-0.14 (-0.23, -0.05)	-0.04 (-0.09, 0.02)	0.22 (0.13, 0.30)
LS Mean Difference (95% CI)	0.68 (0.38, 0.97)	0.64 (0.39, 0.90)	0.37 (0.25, 0.49)	0.36 (0.20, 0.51)
p-value$_{Difference}$	<.0001	<.0001	<.0001	<.0001

Abbreviations: 6M, 6-month follow-up; LS, least squares adjusted; CI, confidence interval; IPW, Inverse Probability Weighting

[a] Adjusted for baseline perceived risk, age, gender, race/ethnicity, education, and company

[b] Screening behaviors included mammography, breast MRI, and clinical breast exam for breast cancer; prostate-specific antigen (PSA) testing for prostate cancer; colonoscopy for colorectal cancer; and unspecified lung cancer screening.

[c] Censoring due to missing data for perceived risk at 6M

[d] Multivariate lung cancer analyses additionally adjusted for smoking status
